# Optimising patient outcomes: temporal trends in remission rates of rheumatoid arthritis patients in the Australian OPAL dataset between 2009 and 2022

**DOI:** 10.1007/s10067-024-06967-8

**Published:** 2024-04-18

**Authors:** Geoffrey Littlejohn, Nithila Anbumurali, Catherine O’Sullivan, Tegan Smith, Kathleen Tymms, Paul Bird, David Nicholls, Hedley Griffiths

**Affiliations:** 1 OPAL Rheumatology Ltd, Sydney, NSW Australia; 2 Monash University, Clayton, VIC Australia; 3 Canberra Rheumatology, Canberra, ACT Australia; 4 University of New South Wales, Kensington, Australia; 5 Emeritus Research, Botany, NSW Australia; 6 Coast Joint Care and University of The Sunshine Coast, Maroochydore, QLD Australia; 7 Barwon Rheumatology, Geelong, VIC Australia; 8 Suite H, Monash Medical Centre, Clayton, VIC 3168 Australia

**Keywords:** b/tsDMARDs, Real-world data, Remission, Rheumatoid arthritis

## Abstract

**Objective:**

To describe the trends in remission rates among RA patients in the OPAL dataset, spanning from 2009 to 2022, and provide insights into the effectiveness of evolving RA management approaches in real-world clinical settings.

**Methods:**

Patients with a physician diagnosis of RA and at least 3 visits between 1 January 2009 and December 2022 were identified in the OPAL dataset, an aggregated collection of data extracted from the electronic medical records of patients managed by 117 Australian rheumatologists. Demographics, disease history, prescribed medications and proportions of patients in Disease Activity Score 28-joint count C-reactive protein (DAS28CRP)) categories (remission, low disease activity (LDA), moderate disease activity (MDA) and high disease activity (HDA)) were described.

**Results:**

A large population (*n* = 48,388) of eligible patients with RA were identified in the OPAL dataset. A consistent and substantial improvement in DAS28CRP remission rates were found in (i) all patients, (ii) patients managed on conventional synthetic disease-modifying antirheumatic drugs (csDMARD) and (iii) patients treated with biological or targeted synthetic (b/ts)DMARD therapy, increasing from approximately 50% in 2009 to over 70% by 2022. The increase in DAS28CRP remission was accompanied by reduced proportions of patients in MDA and HDA states.

**Conclusion:**

This study highlights a consistent improvement in disease activity and rising remission rates among Australian RA patients within the OPAL dataset, offering the potential for enhanced patient outcomes and reduced disease burden.

**Supplementary Information:**

The online version contains supplementary material available at 10.1007/s10067-024-06967-8.

**Table:** 

**Key Points** *• The treat-to-target (T2T) approach, with the treatment goal of remission or, as an alternative, a state of LDA, has been widely adopted for the management of RA in Australia.* *• This study demonstrates an encouraging trend of improved disease activity over time, with remission rates of Australian patients with RA steadily increasing year on year.*		

## Introduction

Despite therapeutic advances and focussed management protocols such as treat-to-target (T2T), not all rheumatoid arthritis (RA) patients achieve remission or optimal disease control. There remains a need to investigate whether remission rates have improved over time with the evolving treatment landscape. Assessing the temporal trends in remission rates is essential to understand the overall effectiveness of current therapeutic approaches and to identify areas where further improvements can be made. This analysis holds particular significance for the OPAL (**O**ptimising **P**atient outcomes in **A**ustralian Rheumato**L**ogy) consortium, as the primary objective has been to optimise patient outcomes. OPAL is a not-for-profit research group that consists of rheumatologists working in predominantly non-hospital clinical practice in Australia [1]. As part of this initiative, the consortium has invested in a cutting-edge electronic medical record (EMR) system called Audit 4 (Software for Specialists), tailored specifically for rheumatology, providing a convenient measurement of disease activity in RA using the Disease Activity Score 28-joint count indices (DAS28). The EMR also prominently displays this information on the patient summary page, facilitating easy access and reference. All data is electronically collected at the point of care.

In 2010, the OPAL Rheumatology group conducted a cross-sectional analysis known as the REMISSION study to assess the disease activity status of patients with RA. The study revealed that a majority of patients achieved remission or had LDA, but a significant number still had moderate (MDA) or high disease activity (HDA) based on the DAS28 erythrocyte sedimentation rate (ESR) categories [2]. A subsequent follow-up study, published in 2015, examined the disease activity status of the OPAL cohort over a 5-year period from 2009 to 2015. This study demonstrated a gradual increase in remission rates according to the DAS28ESR measure, accompanied by a decrease in the proportion of patients with HDA, MDA and LDA [3]. Building upon these earlier investigations, the current study represents another follow-up assessment of remission rates in the OPAL cohort since data collection began in 2009 up until 2022. By examining the remission rates over this extended period, the study aims to provide updated insights into the progress and changes in disease activity among patients with RA treated in Australia.

## Methods

### Study design and setting

This was a retrospective, cohort study describing the disease activity over time in patients with RA in the OPAL dataset. At present, 117 rheumatologists (approximately one-third of Australian rheumatologists) practising in 43 predominantly private clinics around Australia (Queensland, New South Wales, Victoria, Western Australia, Tasmania and the Australian Capital Territory) are contributing their clinical records to the OPAL Rheumatology initiative. All OPAL rheumatologists employ a customised EMR called Audit4, developed by Software 4 Specialists, Pty Ltd, in their regular clinical practice. On a quarterly basis, deidentified clinical data collected at the point of care are extracted from all participating sites and combined to form the OPAL dataset. Data available for this study included patient demographics (age, sex); time from symptom onset to first-recorded visit; RA medications (corticosteroids, conventional synthetic (cs)DMARDs and b/tsDMARDs); disease measures (DAS28-C-reactive protein (CRP), tender joint count (TJC), swollen joint count (SJC)) and acute phase reactants (ESR, and CRP). Data were deidentified to the patient, clinician and clinic prior to extraction from the clinician’s server and aggregated across all sites prior to analysis.

In accordance with the Declaration of Helsinki, the University of New South Wales Human Research Ethics Committee has approved the use of deidentified data captured during routine care in the OPAL dataset for research purposes (HC17799).

### Participants

In Australia, patients with suspected rheumatoid arthritis are referred by their general practitioner to a rheumatologist for definitive diagnosis and management. DMARDs are almost always only initiated by the rheumatologist. Patients in this study were included if they were registered in the OPAL dataset with a diagnosis of RA (ICD-10 code M05. or M06.) made by the rheumatologist, were aged between 18 and 95 years of age and had at least three visits recorded during the study period between 01 January 2009 and 31 of December 2022. The breakdown of the various ICD10 codes (M05 and M06) included can be found in Supplementary Table S1. The date of first symptoms of RA was recorded as the onset of RA by the OPAL rheumatologist, but there are no details relating to a previous established diagnosis by the general practitioner.

### Statistical methods

This study used descriptive statistics for analyses of all endpoints. Numeric variables were described using median, interquartile range (IQR) and range. Categorical and binary variables were described using counts (*n*) and percentages. Percentages of missing data are described. All analyses were performed using R version 4.1.2.

The disease activity categories for DAS28CRP are based on the following cut-offs, remission < 2.6, LDA 2.6 to < 3.2, MDA 3.2 to < 5.1, or HDA ≥ 5.1. For reporting of DAS28CRP, the last recorded DAS28 measure of the corresponding year has been reported. DAS28CRP scores of treated patients were only reported if the csDMARD or b/tsDMARD treatment occurred at the time of the DAS28CRP score.

### Patient involvement

Patients were not involved in the design of this study or consulted during the selection of outcomes or the interpretation of findings. Patients will be involved in the dissemination of this research.

## Results

### Demographics and disease history

A large population (*n* = 48,388) of eligible patients with RA were identified in the OPAL dataset (breakdown of ICD-10 codes for RA diagnoses included in supplementary Table S1). The gender, median age, median time from RA symptom onset to the first visit, median number of visits per year, median time between visits and percentage of patients treated with no DMARD, csDMARD or a b/tsDMARD, for each year, from 2009 to 2022 are provided in Table 1. The same data for patients with at least one DAS28CRP recorded has been provided in supplementary Table S2.

**Table 1:** Demographic and basic clinical features of patients with rheumatoid arthritis (all patients)

**Year**	Number of patients with data available (*n*, %) (*n* = 48388)	Gender—female (*n*, %)	Age at index, years (median (IQR), full range)	Time from RA symptom onset to 1st visit (months) (median (IQR), full range)	Number of visits per patient, per year (median)	Time between visits (median, months)	CCP and/or RF	Treatment (*n*, %)		
								No DMARD	csDMARD	btsDMARD
**2009**	4160 (8.6%)	3100 (74.5%)	60 [51–69], [8–95]	90 [33–180], [− 134–695]	2 [1–3], [1–12]	2 [1–4], [0–10]	1508 (36.2%)	1261 (30%)	2257 (54%)	642 (15%)
**2010**	8869 (18.3%)	6573 (74.2%)	61 [51–69], [9–96]	64 [18–139.5], [− 147–731]	2 [1–3], [1–18]	3 [2 − 5], [0–11]	3144 (35.4%)	2257 (25%)	4682 (53%)	1930 (22%)
**2011**	10938 (22.6%)	8051 (73.8%)	61 [52–70], [10–99]	49 [9–133.75], [− 134–829]	2 [2–4], [1–18]	3 [2–5], [0–11]	4139 (37.8%)	2380 (22%)	5870 (54%)	2688 (25%)
**2012**	12961 (26.8%)	9510 (73.6%)	61 [52–70], [11–112]	28 [4–112], [− 127–826]	2 [2–4], [1–24]	3 [2–5], [0–11]	5193 (40.1%)	2867 (22%)	6741 (52%)	3353 (26%)
**2013**	14626 (30.2%)	10704 (73.4%)	62 [52–70], [12–99]	17 [4–104.75], [− 111–948]	3 [2–4], [1–24]	3 [2–5], [0–11]	5934 (40.6%)	3009 (21%)	7618 (52%)	3999 (27%)
**2014**	15894 (32.8%)	11614 (73.3%)	62 [52–71], [13–115]	12 [3–114], [− 95–840]	2 [2–4], [1–27]	3 [2–5], [0–11]	6638 (41.8%)	3161 (20%)	8111 (51%)	4622 (29%)
**2015**	18410 (38%)	13401 (73.1%)	62 [52–71], [14–116]	24 [5–119.5], [− 89–1021]	3 [2–4], [1–28]	3 [2–5], [0–11]	7663 (41.6%)	3479 (19%)	9150 (50%)	5781 (31%)
**2016**	20304 (42%)	14746 (73%)	63 [53–71], [15–117]	15 [4–84], [− 71–840]	3 [2–4], [1–35]	3 [2–5], [0–11]	8538 (42.1%)	3612 (18%)	9874 (49%)	6818 (34%)
**2017**	21834 (45.1%)	15827 (72.9%)	63 [53–72], [14–118]	19 [4–98], [− 62–1106]	3 [2–4], [1–22]	3 [2–5], [0–11]	9246 (42.3%)	3799 (17%)	10256 (47%)	7779 (36%)
								No DMARD	csDMARD	b/tsDMARD
**2018**	23647 (48.9%)	17100 (72.7%)	63 [53–72], [14–119]	17 [4–94.75], [− 48–694]	3 [2–4], [1–50]	3 [2–5], [0–11]	10,139 (42.9%)	4201 (18%)	10801 (46%)	8645 (37%)
**2019**	24968 (51.6%)	17930 (72.3%)	64 [53–72], [16–119]	13 [4–78.25], [− 37–678]	3 [2–4], [1–26]	3 [2–5], [0–11]	10772 (43.1%)	4277 (17%)	11061 (44%)	9630 (39%)
**2020**	26487 (54.7%)	18890 (72.1%)	64 [53–73], [16–115]	12 [3–67], [− 27–1086]	3 [2–4], [1–37]	3 [2–5], [0–11]	11166 (42.2%)	4652 (18%)	11349 (43%)	10486 (40%)
**2021**	27408 (56.6%)	19484 (71.9%)	64 [54–73], [17–121]	13 [4–72], [− 17–624]	3 [2–4], [1–28]	3 [2–5], [0–11]	11357 (41.4%)	4823 (18%)	11213 (41%)	11372 (41%)
**2022**	26358 (54.5%)	18717 (71.8%)	65 [54–74], [18–122]	12 [4–84], [− 7–660]	3 [2–4], [1–38]	3 [2–5], [0–11]	10969 (41.6%)	4440 (17%)	10169 (39%)	11749 (45%)

*csDMARD* conventional synthetic disease-modifying antirheumatic drugs, b/tsDMARD; biologic and targeted synthetic disease-modifying antirheumatic drugs

As expected, most patients each year were female, constituting approximately 73% of the patient population. The median age of patients in 2009 was 60 years (IQR 51–69), and over the study period, there was an observed increase in the median age of patients, reaching 65 years (IQR 54–74) by 2022. This age increase reflects the length of follow-up in the dataset. The study observed a decreasing trend in the time elapsed from symptom onset to the first visit with an OPAL rheumatologist. Prior to 2011, the median time from symptom onset to the first visit was longer than 50 months, suggesting a significant delay between the onset of RA symptoms and patients receiving specialist care. However, this delay shortened over time, reaching approximately 20 months from 2020 onwards. This indicates an improvement in the timeliness of patients seeking and receiving specialist care for RA in recent years. A shift in the treatment patterns for patients with RA was also observed over the study period. The percentage of patients treated with a b/tsDMARD increased from 15% in 2009 to 45% in 2022. Conversely, the percentage of patients managed on csDMARD therapy decreased from 54% in 2009 to 39% by 2022 (Table 1).

The demographics, disease history and median number of visits per year of patients treated with csDMARDs and patients treated with b/tsDMARDs are given in Table 2. The median number of annual visits for patients managed on csDMARD therapy was two per year, compared to three visits for patients treated with b/tsDMARD therapy. Treatment with corticosteroids has remained relatively stable over time, with around 49% of csDMARD-treated patients and 54% of b/tsDMARDs-treated patients also treated with corticosteroids (Table 2).

**Table 2:** Demographic and basic clinical features of patients with rheumatoid arthritis treated with csDMARD and b/tsDMARD with DAS28CRP data available

	csDMARD-treated patients						b/tsDMARD-treated patients					
Year	Number of patients with data available (*n*, %), (*n* = 48,388)	Gender—female (*n*, %)	Age at index, years (median (IQR), full range)	Number of visits per patient recorded (median)	Corticosteroids (*n*, %)	DAS28-CRP (median)	Number of patients with data available (*n*, %), (*n* = 48,388)	Gender—female (*n*, %)	Age at index, years (median (IQR), full range)	Number of visits per patient recorded (median)	Corticosteroids (*n*, %)	DAS28-CRP (median)
2009	2257 (4.7%)	1658 (73.5%)	61 [52–70], [16–95]	2 [1–11]	997 (44.2%)	2.6 [1.8–3.5], [1–8.2]	642 (1.3%)	483 (75.2%)	58 [49–67], [16–87]	2 [1–4], [1–12]	297 (46.3%)	2.6 [2–3.6], [1–8]
2010	4682 (9.7%)	3449 (73.7%)	62 [52–71], [9–96]	2 [1–3], [1–14]	1893 (40.4%)	2.6 [1.9–3.4], [1–7.8]	1930 (4%)	1435 (74.5%)	59 [49–67], [17–89]	3 [2–5], [1–18]	797 (41.3%)	2.8 [2–3.8], [1–8.2]
2011	5870 (12.1%)	4243 (72.5%)	63 [53–71], [10–97]	2 [2, 3], [1–15]	2578 (43.9%)	2.5 [1.8–3.4], [1–8.4]	2688 (5.6%)	2030 (75.7%)	59 [50–67], [18–90]	3 [2–5], [1–18]	1223 (45.5%)	2.5 [1.9–3.4], [1–7.9]
2012	6741 (13.9%)	4849 (72.1%)	63 [52–71], [11–98]	2 [2, 3], [1–22]	3038 (45.1%)	2.3 [1.7–3.1], [1–8.3]	3353 (6.9%)	2532 (75.7%)	59 [50–67], [14–112]	3 [2–5], [1–24]	1616 (48.2%)	2.4 [1.8–3.3], [1–8.1]
2013	7618 (15.7%)	5480 (72.1%)	63 [53–72], [12–99]	2 [2–4], [1–22]	3610 (47.4%)	2.3 [1.7–3], [1–7.9]	3999 (8.3%)	3023 (75.7%)	60 [50–67], [15–91]	4 [2–5], [1–24]	2062 (51.6%)	2.3 [1.7–3.1], [1–7.9]
2014	8111 (16.8%)	5839 (72.1%)	64 [54–72], [13–99]	2 [2–4], [1–24]	3873 (47.7%)	2.2 [1.6–2.9], [1–7.9]	4622 (9.6%)	3487 (75.7%)	60 [50–68], [16–115]	3 [2–5], [1–27]	2459 (53.2%)	2.3 [1.7–3.1], [1–7.9]
2015	9150 (18.9%)	6551 (71.8%)	64 [54–72], [14–115]	2 [2–4], [1–20]	4524 (49.4%)	2.1 [1.6–3], [1–7.5]	5781 (11.9%)	4337 (75.3%)	60 [50–68], [14–116]	3 [2–5], [1–28]	3090 (53.5%)	2.3 [1.7–3.1], [1–8.1]
2016	9874 (20.4%)	6996 (71.2%)	65 [54–73], [15–117]	2 [1–3], [1–20]	4876 (49.4%)	2.1 [1.6–2.9], [1–8.3]	6818 (14.1%)	5143 (75.7%)	60 [51–69], [15–106]	3 [2–5], [1–35]	3715 (54.5%)	2.2 [1.7–3.1], [1–8.2]
2017	10256 (21.2%)	7230 (70.9%)	65 [55–73], [15–118]	2 [1–3], [1–18]	5122 (49.9%)	2.1 [1.6–3], [1–8.2]	7779 (16.1%)	5830 (75.2%)	61 [51–69], [16–95]	3 [2–5], [1–22]	4261 (54.8%)	2.2 [1.7–3], [1–8.1]
2018	10801 (22.3%)	7602 (70.8%)	65 [55–74], [15–119]	2 [2, 3], [1–18]	5371 (49.7%)	2 [1.6–2.9], [1–8.4]	8645 (17.9%)	6462 (75%)	61 [51–70], [17–113]	3 [2–5], [1–50]	4770 (55.2%)	2.1 [1.6–3], [1–7.8]
2019	11061 (22.9%)	7758 (70.7%)	66 [55–74], [16–119]	2 [2, 3], [1–18]	5503 (49.8%)	2 [1.6–2.8], [1–8.1]	9630 (19.9%)	7151 (74.6%)	62 [52–70], [16–114]	3 [2–5], [1–26]	5369 (55.8%)	2 [1.6–2.9], [1–7.8]
2020	11349 (23.5%)	7916 (70.5%)	66 [55–74], [16–98]	2 [2–4], [1–17]	5668 (49.9%)	1.9 [1.6–2.8], [1–8.1]	10486 (21.7%)	7726 (74.2%)	62 [52–71], [17–97]	3 [2–5], [1–37]	5779 (55.1%)	1.9 [1.6–2.8], [1–8]
2021	11213 (23.2%)	7795 (70.3%)	66 [56–75], [17–121]	2 [2–4], [1–26]	5415 (48.3%)	1.9 [1.5–2.8], [1–7.6]	11372 (23.5%)	8312 (73.7%)	63 [52–71], [18–98]	3 [2–5], [1–28]	6144 (54%)	1.9 [1.6–2.8], [1–7.8]
2022	10169 (21%)	7073 (70.4%)	68 [57–76], [18–122]	2 [2, 3], [1–38]	4895 (48.1%)	1.9 [1.5–2.7], [1–7.7]	11749 (24.3%)	8565 (73.6%)	63 [53–72], [18–101]	3 [2–5], [1–23]	6399 (54.5%)	1.9 [1.6–2.7], [1.1–7.8]

*csDMARD* conventional synthetic disease-modifying antirheumatic drugs, b/tsDMARD; biologic and targeted synthetic disease-modifying antirheumatic drugs

### Disease activity

**Fig. 1:**
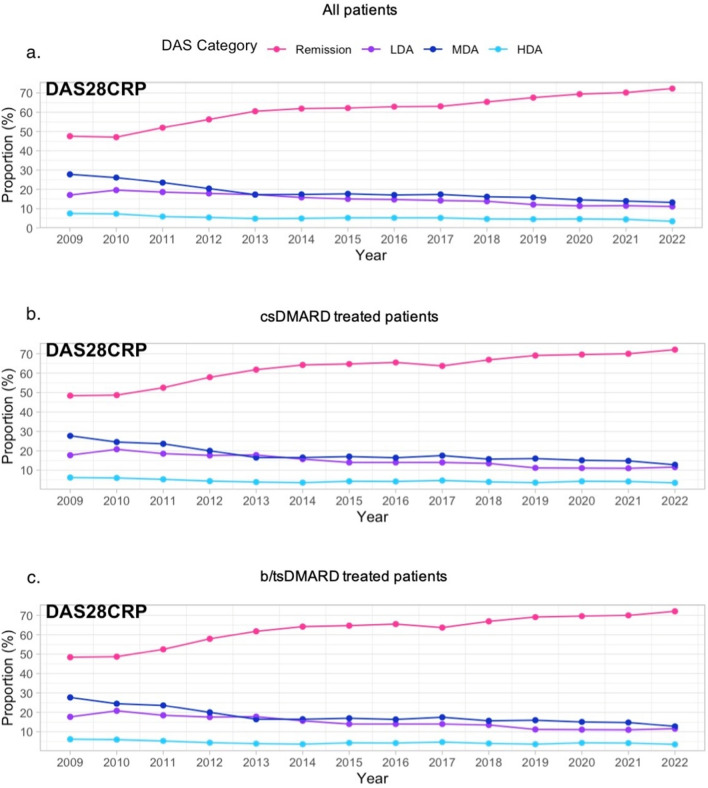
Proportion of patients at each DAS28CRP category for all patients (**a**), csDMARD-treated patients (**b**) and b/tsDMARD-treated patients (**c**). csDMARD conventional synthetic disease-modifying antirheumatic drugs, b/tsDMARD biological or targeted synthetic disease-modifying antirheumatic drugs, LDA low disease activity, MDA moderate disease activity, HDA high disease activity

Disease activity was evaluated in all patients plus patients managed with csDMARD and b/tsDMARD therapy. The median DAS28CRP score for all three groups was 2.6 in 2009, decreasing to 1.9 in 2022 (Table 2). A consistent and notable trend of increasing annual remission rates and decreasing proportions of patients in MDA and HDA can also be seen over time in all three groups (Figs. 1a, b and c, respectively). In 2009, just under 50% of patients were in DAS28CRP remission, increasing to over 70% in 2022. The proportion of patients in MDA decreased from ~ 28 to 13%, and the proportion of patients in HDA decreased from ~ 7.5% in 2009 to 3.5% in 2022 for all patients, csDMARD-treated and for b/tsDMARD-treated patients (Fig. 1). These findings collectively demonstrate a substantial and consistent improvement in disease activity control, characterised by increasing remission rates and decreasing proportions of patients in both MDA and HDA states, across all patient groups from 2009 to 2022.

**Fig. 2:**
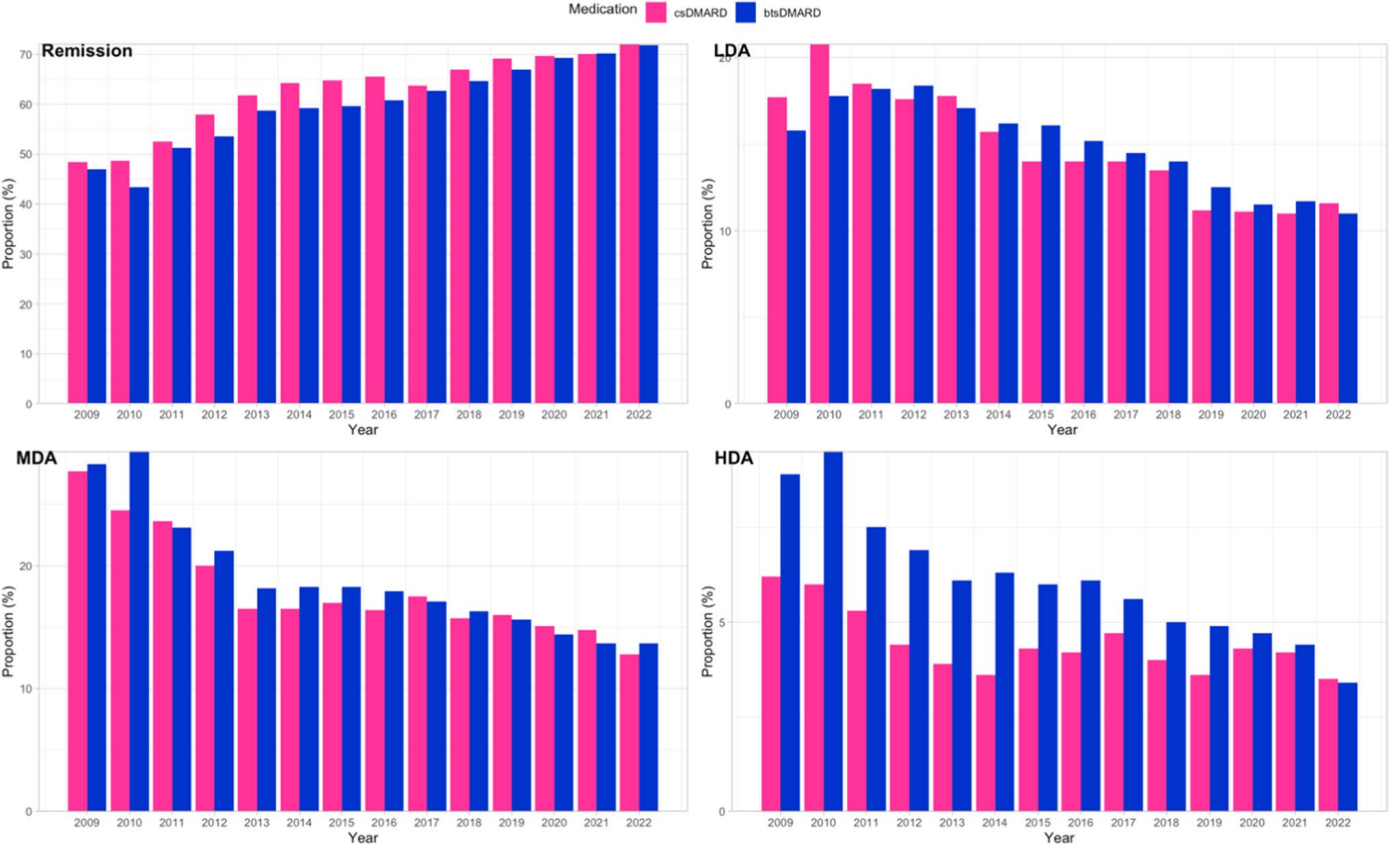
Proportion of csDMARD- and b/tsDMARD-treated patients at each DAS28CRP category. csDMARD conventional synthetic disease-modifying antirheumatic drugs, b/tsDMARD biological or targeted synthetic disease-modifying antirheumatic drugs, LDA low disease activity, MDA moderate disease activity, HDA high disease activity

The proportion of csDMARD- and b/tsDMARD-treated patients in each DAS28CRP category is depicted in Fig. 2, demonstrating a similar trend of increasing remission rates and decreasing LDA, MDA and HDA over time for patients managed by either csDMARD therapy or b/tsDMARD therapy. While remission, LDA and MDA rates were similar for both csDMARD- and b/tsDMARD- treated groups each year, a higher proportion of b/tsDMARD-treated patients were in HDA than csDMARD-treated patients at the earlier time points. It is important to note that patients in Australia can only access government-subsidised b/tsDMARDS if they continue to have predesignated high disease activity after a 6-month course of a combination of two csDMARDs, including over 20 mg per week of methotrexate. Thus, all b/tsDMARD patients in this study have failed csDMARDs. Additionally, many patients on b/tsDMARDs remain on csDMARDs as concomitant therapy to enable optimum disease control.

### Impact of CCP/RF status, gender, and symptom duration

**Fig. 3:**
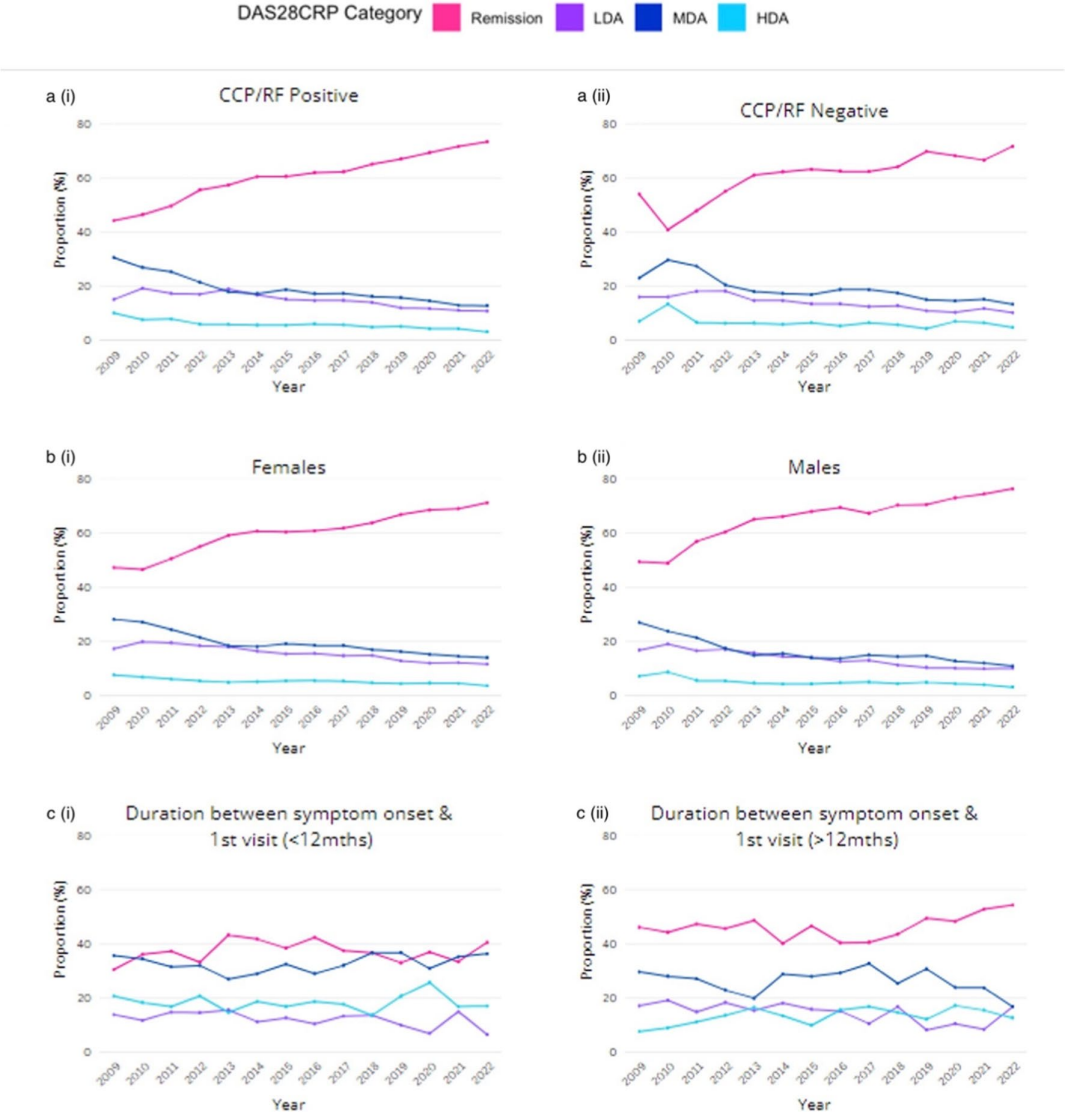
Proportion of patients at each DAS28CRP category for CCP/RF positive and CCP/RF negative patients (**a**), female and male gender (**b**) and symptom duration (**c**). CCP anti-cyclic citrullinated peptide, RF rheumatoid factor, LDA low disease activity, MDA moderate disease activity, HDA high disease activity

The proportion of patients in each DAS28CRP category per year was also assessed for (i) patients categorised as positive or negative for anti-cyclic citrullinated peptide (CCP) and rheumatoid factor (RF), (ii) females and males and (iii) patients with a short duration (≤ 12 months) of symptoms prior to first visit with an OPAL rheumatologist and those with a longer duration (> 12 months) of symptoms prior to first visit (Fig. 3a, b and c, respectively). No differences were observed in remission, LDA, MDA or HDA rates between the CCP/RF positive or negative groups nor between females and males. Notably, patients with a symptom duration of less than 12 months before their first visit consistently displayed a higher median DAS28CRP score each year, with an average score exceeding 3. In contrast, patients reporting symptoms for more than 12 months before their first visit typically had median DAS28CRP scores ranging between 2.5 and 2.7 each year. Furthermore, patients who experienced a shorter duration between the onset of symptoms and their first recorded visit showed a comparatively modest increase in remission rates, which escalated from 30.4% in 2009 (*n* = 102) to 40.4% in 2022 (*n* = 94) (Fig. 3ci). Conversely, patients with a more extended period of symptom onset prior to their first visit, began with a higher percentage in remission, at 46% in 2009 (*n* = 347), rising to 54.2% in 2022 (*n* = 96). It is worth highlighting that individuals with a shorter symptom duration consistently demonstrated elevated rates of MDA throughout the study period. It is important to exercise caution when interpreting these findings due to the limited sample size of patients with available symptom duration data. We note that delay between symptom onset and initiation of DMARDs may affect disease outcomes.

## Discussion

Early diagnosis, prompt treatment and attaining early remission have been linked to significantly improved long-term prognosis for patients with RA [4]. Our study describes the temporal trends in remission rates among RA patients in the Australian OPAL dataset and aims to provide insight into whether evolving approaches to RA management are effectively translating into improved patient outcomes in everyday clinical settings. The OPAL EMR incorporates a user-friendly T2T framework for the busy clinician to measure relevant clinical disease activity and inflammatory indices to enhance management strategies.

We observed several key findings. Notably, the duration from the onset of symptoms to the initial consultation with an OPAL rheumatologist was seen to decrease over time, suggesting improved timeliness in access to specialist care. Moreover, a notable shift in treatment strategies was evident, characterised by a rise in the utilisation of b/tsDMARDs in later years, accompanied by a concurrent decrease in csDMARDs as the main treatment strategy. These trends collectively suggest earlier access to rheumatological expertise, facilitating swifter diagnoses and aligning with the availability of newer b/tsDMARDs over time, as factors contributing to more effective therapeutic management of the disease. It is important to note that it is not known if the first visit recorded in the OPAL dataset was the patient’s first encounter with a rheumatologist for their RA symptoms. It is possible that a patient had previously been seen by another specialist or changed rheumatologists during their treatment journey. This study only included deidentified data that was derived from the EMR of clinicians contributing to OPAL. It is noted therefore that the contribution of the time taken to achieve LDA/remission after the initiation of DMARDs is not known in this study.

Consequently, we found a consistent trend of improved disease activity over time with remission rates increasing from approximately 50% in 2009 to over 70% by 2022, with a concomitant decrease in the proportion of patients in MDA and HDA. This trend was seen for all patient groups whether they were managed with csDMARD therapy or b/tsDMARD therapy. The contribution of b/tsDMARDs to the high remission rates in this group requires further well-designed studies. However, it can be speculated that these enhanced remission rates would translate into a reduced disease burden and fewer hospital admissions and improved disease management.

An increase in remission rates over the years has also been reported in other registries [5–7]. In a large-scale multinational study in 11 countries in the Asia–Pacific region, an overall DAS28CRP remission rate of 62.3% was reported; however, it should be noted that the remission rates varied greatly between countries, ranging from 13.9 to 77.1% [7].

The percentage of patients receiving no DMARD at the time of each data cut, as shown in Table 1, fell from 30 to 17% over the study period. These patients are not well characterised in this study but likely represent patients managed only on low-dose steroids or with otherwise mild or no disease activity at the time of assessment.

The impact of CCP and RF on disease activity remains a subject of debate within the literature. While certain studies suggest that positive CCP and RF are correlated with increased disease activity and radiographic progression, others propose an association between positive CCP and a lower degree of clinical disease activity [8–10]. In the Ontario Best Practices Research Initiative registry, combined CCP and RF positivity were found to be linked to a higher remission rate and greater improvement in disease activity when undergoing antirheumatic medication treatment [10]. In the present study, no differences in remission rates were observed between the CCP- and/or RF-positive patients compared to CCP/RF-negative patients. Previous studies have associated the male sex with a higher likelihood of remission, including sustained remission, but other studies have not [11]. This association was not apparent in our study, as a similar proportion of females and males were in DAS28CRP remission throughout the years (reaching 71% of females and 76.1% of males in remission in 2022).

The proportion of patients receiving corticosteroids in addition to their csDMARD or b/tsDMARD therapy has risen very slightly in both cohorts over the study period. Specifically, in the csDMARD-managed group, this figure increased from 44.2% in 2009 to 48.1% in 2022, while in the b/tsDMARD-managed group, it rose from 46.3% in 2009 to 54.5% in 2022. Unfortunately, this dataset lacks information regarding the corticosteroid dosage, and it remains unclear whether the recorded steroid use in the prescribing section of the EMR is current or if the treatment has simply not been discontinued.

While b/tsDMARDs have proven highly effective in reducing inflammation and making clinical remission an attainable goal, there remains a subset of patients for whom achieving remission or LDA remains elusive.

## Conclusion

In conclusion, our study has demonstrated an encouraging trend of improved disease activity over time, with remission rates of Australian patients with RA steadily increasing year on year. These rising remission rates hold the potential to translate into fewer hospital admissions and a reduced disease burden among the Australian population.

## Limitations

This was a retrospective, observational study of a dataset comprising clinical data captured for the primary purpose of routine clinical care. As a result, missing data for various outcome variables was observed. Although the concept of remission has been accepted, there is currently no standard definition that has been universally agreed upon to define it. The current study utilised the DAS28CRP definition of remission, but there are also the ACR/European Alliance of Associations for Rheumatology (EULAR) Boolean-based remission definition, DAS28 erythrocyte sedimentation rate (ESR), Simplified Disease Activity Index (SDAI) and Clinical Disease Activity Index (CDAI), making it difficult to compare across studies.

The study did not assess confounding factors such as delay in referral, treatment duration, concomitant steroid use, treatment choice or specific cs/b/tsDMARD combinations. As such, no conclusions can be made of the contribution of b/tsDMARDs to the outcomes per se. Rather there are likely many factors contributing to better outcomes, including a larger therapeutic “toolbox” and a more focussed goal-orientated management algorithm.

### Supplementary Information

Below is the link to the electronic supplementary material.

Supplementary file1 (DOCX 24 KB)

## Data Availability

Data collection is based on opt-out patient consent, and patients have consented to their data being made available to OPAL Rheumatology only. Requests for access to summary statistics will be considered by the OPAL Scientific Review Committee.
