# Supplementary material for: Optimising patient outcomes: temporal trends in remission rates of rheumatoid arthritis patients in the Australian OPAL dataset between 2009 and 2022
Source: Clin Rheumatol. 2024 Apr 18;43(6):1823–32. doi: 10.1007/s10067-024-06967-8 (PMC11111498; doi:10.1007/s10067-024-06967-8)
Supplement: Supplementary file 1 — Supplementary file1 (DOCX 24 KB) [file 10067_2024_6967_MOESM1_ESM.docx]

**Optimising patient outcomes: temporal trends in remission rates of rheumatoid arthritis patients in the Australian OPAL dataset between 2009-2022**

Geoffrey Littlejohn^1,2^, Nithila Anbumurali^1^, Catherine O’Sullivan^1^, Tegan Smith^1^, Kathleen Tymms^1,3^, Paul Bird^1,4,5^, David Nicholls^1,6^, Hedley Griffiths^1,7^

1. OPAL Rheumatology Ltd, Sydney, NSW, Australia
2. Monash University, Clayton, VIC, Australia
3. Canberra Rheumatology, Canberra, ACT Australia
4. University of New South Wales, Kensington, Australia
5. Emeritus Research, Botany, NSW, Australia
6. Coast Joint Care and University of The Sunshine Coast, Maroochydore, QLD, Australia
7. Barwon Rheumatology, Geelong, VIC, Australia

Corresponding author: Nithila Anbumurali

Address: c/o OPAL Rheumatology Ltd

Email: [nithila.anbu@opalrheumatology.com.au](mailto:nithila.anbu@opalrheumatology.com.au)

**Journal: Clinical Rheumatology**

**Supplementary File**

Supplementary Table S1: ICD10 codes comprising rheumatoid arthritis patient groups

| ICD10 Code | Description | RA   (n=48388) |
| --- | --- | --- |
| M06.9 | Felty's syndrome | 61 (0.1%) |
| M06.4 | Rheumatoid lung disease | 57 (0.1%) |
| M05.0 | Rheumatoid vasculitis | 14 (0%) |
| M06.3 | Seropositive rheumatoid arthritis | 62 (0.1%) |
| M05.9 | Seronegative rheumatoid arthritis | 60 (0.1%) |
| M05.2 | Adult-onset Still's disease | 76 (0.2%) |
| M05.1 | Rheumatoid nodule(s)/Rheumatoid nodulosis due to methotrexate | 149 (0.3%) |
| M06.0 | Inflammatory polyarthropathy/Seronegative arthropathy | 11781 (24.3%) |
| M06.1 | Other specified rheumatoid arthritis | 6 (0%) |
| M06.8 | Rheumatoid arthritis | 37898 (78.3%) |

Supplementary Table S2: Demographics, disease history and visit frequency of patients with rheumatoid arthritis with at least one DASCRP

|  | Number of patients with data available (n, %) (n=48388) | Gender - Female (n, %) | Age at Index, years (median [IQR], full range) | Time from RA symptom onset to 1^st^ visit in year (months) (median [IQR], full range) | Duration of care recorded in Audit4 at 1st visit in year (years) (median [IQR], full range) | Number of visits per patient recorded (median) | Time between visits (median, months) | CCP and/or RF | Treatment (n, %) | | |
| --- | --- | --- | --- | --- | --- | --- | --- | --- | --- | --- | --- |
|  |  |  |  |  |  |  |  |  | No DMARD | csDMARD | b/tsDMARD |
| 2009 | 1234 (2.6%) | 911 (73.8%) | 61 [52-69],[16-90] | 93 [34-186],[-2-695] | 0 [0-0],[0-9] | 2 [1-3],[1-12] | 2 [2-4],[0-10] | 477 (38.7%) | 80 (6%) | 858 (70%) | 296 (24%) |
| 2010 | 3256 (6.7%) | 2361 (72.6%) | 62 [52-70],[19-96] | 76 [27-146],[-108-731] | 0 [0-1],[0-10] | 3 [2-4],[1-18] | 3 [2-5],[0-10] | 1318 (40.5%) | 120 (4%) | 2095 (64%) | 1041 (32%) |
| 2011 | 4619 (9.5%) | 3370 (73.1%) | 62 [52-70],[16-97] | 51 [12-140],[-4-810] | 1 [0-2],[0-11] | 3 [2-4],[1-18] | 3 [2-5],[0-11] | 2008 (43.5%) | 165 (4%) | 2946 (64%) | 1508 (33%) |
| 2012 | 4967 (10.3%) | 3615 (73%) | 61 [52-70],[15-112] | 23 [4.5-105],[-5-720] | 2 [1-2],[0-12] | 3 [2-4],[1-24] | 3 [2-5],[0-11] | 2335 (47%) | 192 (4%) | 2960 (60%) | 1815 (37%) |
| 2013 | 5756 (11.9%) | 4174 (72.7%) | 62 [52-70],[17-94] | 30.5 [6-144],[-5-948] | 2 [1-3],[0-13] | 3 [2-5],[1-24] | 3 [2-5],[0-11] | 2800 (48.6%) | 159 (3%) | 3299 (57%) | 2298 (40%) |
| 2014 | 6156 (12.7%) | 4527 (73.7%) | 62 [52-70],[18-115] | 24 [5-168],[-5-840] | 3 [1-4],[0-14] | 3 [2-5],[1-27] | 3 [2-5],[0-11] | 3131 (50.9%) | 142 (2%) | 3264 (53%) | 2750 (45%) |
| 2015 | 7138 (14.8%) | 5219 (73.4%) | 62 [53-70],[17-115] | 40 [7-135],[-2-860] | 3 [1-5],[0-15] | 3 [2-4],[1-28] | 3 [2-5],[0-11] | 3619 (50.7%) | 163 (2%) | 3603 (50%) | 3372 (47%) |
| 2016 | 7146 (14.8%) | 5227 (73.5%) | 62 [52-70],[18-106] | 23 [5.25-110],[-71-840] | 4 [1-6],[0-16] | 3 [2-4],[1-35] | 3 [2-5],[0-11] | 3697 (51.7%) | 152 (2%) | 3303 (46%) | 3691 (52%) |
|  |  |  |  |  |  |  |  |  | No DMARD | csDMARD | b/tsDMARD |
| 2017 | 7241 (15%) | 5305 (73.6%) | 62 [53-71],[18-95] | 28.5 [5-149],[-3-660] | 4 [2-7],[0-17] | 3 [2-4],[1-21] | 3 [2-5],[0-11] | 3775 (52.1%) | 142 (2%) | 3020 (42%) | 4079 (56%) |
| 2018 | 7586 (15.7%) | 5534 (73.3%) | 63 [53-71],[16-108] | 16 [4-120.25],[-9-689] | 5 [2-8],[0-18] | 3 [2-4],[1-32] | 3 [2-5],[0-11] | 4095 (54%) | 138 (2%) | 3008 (40%) | 4440 (59%) |
| 2019 | 7738 (16%) | 5658 (73.5%) | 63 [53-71],[17-97] | 24 [5-137.25],[-10-677] | 5 [2-8],[0-19] | 3 [2-4],[1-26] | 4 [2-6],[0-11] | 4229 (54.7%) | 164 (2%) | 2763 (36%) | 4811 (62%) |
| 2020 | 7097 (14.7%) | 5197 (73.7%) | 63 [53-72],[17-97] | 19 [4-120],[-7-600] | 6 [3-9],[0-20] | 3 [2-4],[1-21] | 3 [2-6],[0-11] | 3843 (54.1%) | 177 (2%) | 2168 (31%) | 4752 (67%) |
| 2021 | 7741 (16%) | 5645 (73.4%) | 64 [53-72],[18-99] | 24 [6-120],[-1-624] | 6 [3-10],[0-21] | 3 [2-4],[1-21] | 3 [2-5],[0-11] | 4238 (54.7%) | 220 (3%) | 2283 (29%) | 5238 (68%) |
| 2022 | 7762 (16%) | 5647 (73.3%) | 64 [54-73],[18-98] | 13 [5-88.75],[-3-520] | 7 [3-11],[0-22] | 3 [2-4],[1-23] | 4 [2-5],[0-11] | 4164 (53.6%) | 278 (4%) | 2212 (28%) | 5272 (68%) |
